# Distribution of High-Risk HPV in Cervical Versus Oropharyngeal Samples: Results from a Prospective Screening Study in a Cohort of European Women of Reproductive Age

**DOI:** 10.3390/pathogens14121274

**Published:** 2025-12-11

**Authors:** Dagmara Lisman, Andrzej Ossowski, Bartłomiej Grygorcewicz, Łukasz Tyszler, Rafał Becht, Mateusz Kozłowski, Aneta Cymbaluk-Płoska

**Affiliations:** 1 Department of Genomics and Forensic Genetics, Pomeranian Medical University in Szczecin, 70-111 Szczecin, Polandbartlomiej.grygorcewicz@pum.edu.pl (B.G.); 2 Department of Conservative Dentistry and Endodontics, Faculty of Dentistry, Pomeranian Medical University in Szczecin, 70-204 Szczecin, Poland; 3 Clinical Department of Oncology, Chemotherapy and Cancer Immunotherapy, Pomeranian Medical University in Szczecin, 70-204 Szczecin, Poland; 4 Department of Reconstructive and Oncological Gynecology, Pomeranian Medical University in Szczecin, 70-204 Szczecin, Poland; mateusz.kozlowski@pum.edu.pl (M.K.);

**Keywords:** human papillomavirus (HPV), high-risk HPV, cervical cancer screening, oropharyngeal samples

## Abstract

High-risk human papillomavirus (HR-HPV) infection with genotypes such as HPV-16 and HPV-18 is a well-established risk factor for cervical cancer; however, its prevalence in oropharyngeal sites among asymptomatic women remains less clearly defined. We evaluated 400 women aged 20–40 years (mean age 29.6 years) for the presence of HPV-16, HPV-18, and other HR-HPV genotypes in both cervical and oropharyngeal samples. Cervical specimens were collected using flocked swabs, and oropharyngeal specimens using flocked nylon swabs. Samples were preserved in transport medium, transported at 4–8 °C, and stored at –20 °C until DNA extraction. Viral DNA was isolated with the AA Viral DNA Kit and analyzed using the GenoProf HPV Screening Test, which employs multiplex PCR and reverse hybridization. HPV-16 was detected in 9.5% of participants, HPV-18 in 7.3%, and other HR-HPV genotypes in 14.3%. No statistically significant variation in prevalence across age groups was observed (HPV-16: χ^2^ = 0.10, *p* = 0.992; HPV-18: χ^2^ = 0.10, *p* = 0.992; HR-HPV: χ^2^ = 0.15, *p* = 0.985). Importantly, no HPV DNA was detected in oropharyngeal swabs. These findings indicate that, in this cohort of reproductive-age women, HR-HPV infection was confined to the cervix, with oropharyngeal infection being rare or undetectable. The results underscore the importance of prioritizing cervical screening initiatives in similar populations.

## 1. Introduction

Human papillomavirus (HPV) is the most common sexually transmitted viral infection and the necessary cause of nearly all cervical cancers. High-risk genotypes—particularly HPV16 and HPV18—account for the majority of invasive cervical cancers worldwide, with recent IARC analyses estimating that these two types together are responsible for approximately three-quarters of cases across global regions [1]. HPV vaccination programs and HPV-based molecular screening therefore form the cornerstone of contemporary cervical cancer prevention strategies endorsed by the WHO [2].

HPV infections are most extensively studied in the cervical epithelium, where the virus targets the transformation zone composed of actively dividing basal cells, promoting persistent replication and elevating the risk of neoplastic progression [3]. In contrast, HPV prevalence in the oral cavity and oropharynx is substantially lower. Large population-based studies report oral/oropharyngeal HPV DNA detection rates of only 0.5–2% in women, with HPV16 as the most frequently identified genotype [4]. The biological structure of the oropharyngeal mucosa differs significantly from the cervical transformation zone: keratinized and non-keratinized squamous epithelia in the mouth and throat provide limited access to basal cells, epithelial turnover is slower, and the microenvironment is less conducive to sustained HPV persistence [5,6]. As a result, oral HPV infections tend to be transient, characterized by low viral loads and frequently falling below the detection threshold of molecular assays [7].

Beyond the cervix, HPV—especially HPV16—is a recognized etiological factor for a subset of head and neck cancers, most notably oropharyngeal squamous cell carcinoma (OPSCC). Incidence rates of HPV-positive OPSCC have risen sharply in many high-income countries, and the distinct molecular and clinical characteristics of HPV-driven disease are now well established [8]. However, epidemiological research in the oropharyngeal region has focused primarily on men or on patients with established head and neck malignancies [9,10,11]. Consequently, the baseline prevalence of high-risk HPV in the oropharynx of asymptomatic women from the general population remains poorly defined.

This knowledge gap is particularly prominent in Central and Eastern Europe, where robust data on oral/oropharyngeal HPV among healthy women are scarce, and where regional variability in HPV prevalence and vaccination uptake further complicates epidemiologic assessment [12,13,14,15]. Moreover, the expansion of HPV vaccination programs increases the importance of characterizing genotype distribution across different anatomical sites to understand the broader impact of immunization on non-cervical HPV transmission.

Women aged 20–40 years represent a key demographic for HPV epidemiology: this age group experiences the highest incidence of new cervical HPV infections, is typically sexually active, and is within the age range targeted for cervical screening and post-vaccination monitoring [16]. Yet, the dynamics of HPV infection in the oropharynx within this population remain unclear.

From a methodological standpoint, very few studies employ paired sampling from cervical and oropharyngeal sites within the same individuals. Such a design provides a unique opportunity to assess anatomical site-specific differences in HPV prevalence, evaluate potential concordance between sites, and generate internally consistent epidemiological estimates. However, oropharyngeal sampling poses technical challenges: the viral load is often extremely low, sampling methods lack standardization, and results may be affected by variability in oral microbiota or mucosal keratinization [17,18,19]. These factors contribute to inconsistent findings across studies and highlight the need for rigorously designed, population-based investigations.

Therefore, the aim of this study was to evaluate the prevalence and age distribution of HPV16, HPV18, and other high-risk HPV genotypes in paired cervical and oropharyngeal samples from sexually active women aged 20–40 years in Central Europe, providing site-specific data directly relevant to screening practice, infection dynamics, and regional HPV epidemiology.

## 2. Material and Methods

### 2.1. Study Population

In 2025, a total of 400 women aged 20–40 years were enrolled from the West Pomeranian Voivodeship (north-western Poland). The study population consisted of clinically healthy, sexually active women participating in a regional cervical cancer screening program. Information on prior cervical dysplasia, malignancy, or HPV vaccination status was not collected, as participants were recruited within the framework of routine screening examinations. All participants underwent HPV testing based on paired samples collected from the cervix and the oropharynx (posterior pharyngeal wall and palatine tonsils), allowing for a comparative analysis of viral prevalence across genital and extragenital sites.

Eligibility required provision of written informed consent, and all participants were informed about the aims and procedures of the study. The study protocol received a favorable opinion from the Bioethics Committee of the Pomeranian Medical University in Szczecin (approval no. KB-006/25/2025). This study was funded by the West Pomeranian Voivodeship, which supported the research activities and laboratory analyses.

#### 2.1.1. Demographic Characteristics of the Study Population

A total of 400 women aged 20–40 years (mean age 29.6, SD 4.2) were included in the study. The median age of sexual initiation was 17.8 years (range 14–23). The mean cumulative tobacco exposure was 1.8 pack-years (range 0–25.8) (Table 1).

#### 2.1.2. Demographic and Clinical Characteristics

With respect to demographic factors, the study population was balanced across age categories. The majority of women resided in urban areas, with smaller proportions from rural locations. Most participants reported having completed secondary or higher education. In terms of relationship status, women were divided between single, stable partnership, and married categories (Table 1).

Regarding socioeconomic and lifestyle variables, the majority of women reported middle economic status, with smaller groups indicating low or high status. About one third of participants reported a history of smoking, while alcohol use was less frequently declared. The number of lifetime sexual partners varied, but the largest proportion reported 2–4 partners (Table 1).

From a gynecological perspective, approximately half of the women were nulliparous, while the remainder reported one or more births. Use of hormonal contraception was common, and a subset of participants reported intrauterine device (IUD) use. A minority of women declared a history of cervical intraepithelial neoplasia (CIN, LSIL, or HSIL) (Table 1).

HPV vaccination coverage in the study group was limited: many women remained unvaccinated, while others had received partial or complete vaccination, with different vaccine types (bivalent, quadrivalent, or 9-valent) represented.

### 2.2. Methods—Cervical Sample Collection for HPV Testing

Cervical samples were obtained by experienced gynecologists during a routine speculum examination. After visualization of the cervix, excess vaginal mucus was gently removed with a sterile swab to avoid sample contamination. A cervical flocked swab was then inserted into the endocervical canal and rotated 360° for 5–10 s to collect exfoliated epithelial cells from both the ectocervix and endocervical canal.

The brush head was immediately placed into a vial containing liquid-based cytology medium (transport medium), ensuring that the cellular material was fully immersed. Samples were labeled with a unique patient identifier and stored at 4–8 °C during transport. Subsequently, aliquots were frozen at −20 °C until DNA extraction.

All procedures were performed under aseptic conditions, following institutional protocols and international guidelines for cervical cancer screening and HPV DNA testing.

To exclude any technical causes of false-negative results, all oropharyngeal samples underwent a multilevel analytical quality control procedure, including an internal human β-globin control confirming successful amplification and absence of PCR inhibitors, extraction controls (positive spike-in and negative extraction controls), as well as transport sentinel controls processed in parallel with the clinical samples to verify sample integrity and stability throughout the workflow.

### 2.3. Methods—Oropharyngeal Sample Collection for HPV Testing

Oropharyngeal specimens were collected by trained clinicians using a flocked nylon swab (Copan FLOQSwabs^®^, Brescia, Italy). Each participant was instructed to open the mouth widely and phonate “ah” to elevate the soft palate and expose the oropharyngeal structures.

In this study, a throat swab was used as an added value to the project, serving as a screening tool for HPV infections within the upper respiratory tract. This approach enabled the comparison of viral presence across different anatomical sites and allowed for the assessment of the diagnostic potential of this technique in population-based studies. The results confirmed that HPV can be detected in non-neoplastic throat tissue during active infection, indicating the possibility of early infection detection and expanding the understanding of extragenital sites of viral replication [20,21,22].

The swab was inserted into the oral cavity without touching the tongue, teeth, or buccal mucosa, and gently rubbed across the posterior pharyngeal wall and the surface of both palatine tonsils with a rotating motion for approximately 5–10 s. This ensured adequate sampling of exfoliated epithelial cells potentially harboring HPV DNA.

Samples were collected using cervical swabs placed in Universal Transport Medium (UTM™, Copan Diagnostics, Brescia, Italy), which is validated for HPV DNA preservation and recommended for use with the GeneProof HPV PCR assay. Samples were labeled with unique patient identifiers and maintained at 4–8 °C during transport. Upon arrival at the laboratory, specimens were frozen at −20 °C until DNA extraction.

All procedures were conducted under aseptic conditions, following institutional biosafety standards and established protocols for oropharyngeal HPV testing.

### 2.4. Methods—Viral DNA Extraction

DNA was extracted from cervical and oropharyngeal swabs using the AA Viral DNA Kit (A&A Biotechnology, Lancaster, UK), following the manufacturer’s protocol. Briefly, clinical specimens preserved in transport medium were thawed at room temperature and vortexed for homogenization.

An aliquot of 200 µL of each sample was mixed with lysis buffer containing chaotropic salts and proteinase K, followed by incubation at 56 °C for 10–15 min to ensure complete viral capsid disruption and protein digestion.

The lysate was then transferred onto a silica spin column and centrifuged, allowing viral DNA to bind to the membrane. After sequential washing steps to remove residual proteins, salts, and inhibitors, the DNA was eluted in 50 µL of nuclease-free elution buffer.

Extracted DNA was stored at −20 °C until further molecular analysis. For reliable HPV detection, the critical parameter is the number of viral copies present in the clinical specimen rather than the absolute DNA concentration expressed in ng/µL. The analytical sensitivity of most commercial assays corresponds to approximately 10^2^–10^3^ HPV DNA copies per milliliter, which is typically achieved with adequately collected swabs and proper nucleic acid extraction, which is consistent with the expected yield from well-collected clinical swabs and optimized DNA extraction procedures.

### 2.5. Methods—HPV DNA Amplification and Genotyping

Detection and genotyping of HPV DNA were performed using the GenoProf HPV Screening Test (Brno, Czech Republic), according to the manufacturer’s instructions. The assay is based on multiplex PCR amplification of the L1 gene region of the HPV genome, combined with reverse hybridization for genotype-specific identification. The panel enables identification of the most clinically relevant high-risk (HR) HPV genotypes, including: HPV16, HPV18, HPV31, HPV33, HPV35, HPV39, HPV45, HPV51, HPV52, HPV56, HPV58, HPV59, HPV66, and HPV68. In addition, the test also detects several low-risk (LR) genotypes associated with benign lesions, such as HPV6, HPV11, HPV42, HPV43, and HPV44.

The GenoProof HPV Screening Test does not specify or require a minimal DNA concentration expressed in ng/µL, as it is a qualitative RT-PCR assay. The analytical sensitivity is defined in IU/mL (limit of detection), rather than by DNA mass. According to the manufacturer’s instructions, 10 µL of purified nucleic acid should be added to the reaction mixture (40 µL total). Sample adequacy is verified by the endogenous internal control (GAPDH); successful amplification of this control indicates that the amount and quality of the extracted material are sufficient.

Although the GeneProof HPV PCR Kit is primarily validated for cervical samples collected in liquid-based cytology media, PCR-based HPV genotyping assays have been successfully applied to oropharyngeal swabs in several studies, provided that sample quality and amplification controls are ensured [23,24]. It should be noted that these analyses were performed using different diagnostic assays than the one currently in use. This allows comparative analysis of HPV prevalence between cervical and oropharyngeal anatomical sites within the same study population.

Briefly, 10 µL of extracted viral DNA was added to the PCR master mix containing HPV-specific primers, nucleotides, buffer, and Taq DNA polymerase.

PCR products were then subjected to reverse hybridization on a membrane strip coated with probes specific for 14 high-risk (including HPV16 and HPV18) and 6 low-risk HPV genotypes. After stringent washing, bound amplicons were visualized by colorimetric detection, and genotypes were interpreted using the reference guide supplied with the kit.

Positive and negative controls provided in the kit were included in each run to ensure the reliability of amplification and hybridization.

## 3. Results

A total of 400 women aged 20–40 years were included in the study. The mean age of participants was 29.6 years (SD ± 5.4). All women provided paired cervical and oropharyngeal samples.

Table 2 summarizes the prevalence of HPV16, HPV18, and other high-risk HPV (HR-HPV) genotypes across age groups. Overall, HPV16 was detected in 9.5% of cervical samples, HPV18 in 7.3%, and other HR-HPV genotypes in 14.3%. No HPV DNA was detected in oropharyngeal samples, indicating that all infections identified in this cohort were confined to the cervical epithelium.

Association with Age

The distribution of HPV positivity did not differ significantly across age groups:

HPV16: χ^2^ = 0.10, df = 3, *p* = 0.992;

HPV18: χ^2^ = 0.10, df = 3, *p* = 0.992;

HPV_HighRisk: χ^2^ = 0.15, df = 3, *p* = 0.985.

No age-related trends were observed.

The distribution of HPV positivity across age groups did not reveal any statistically significant associations: HPV16 (χ^2^ = 0.10, df = 3, *p* = 0.992), HPV18 (χ^2^ = 0.10, df = 3, *p* = 0.992), and HR-HPV (χ^2^ = 0.15, df = 3, *p* = 0.985).

Importantly, none of the patients tested positive for HPV DNA in throat swabs, confirming that all identified infections were localized to the genital tract.

Demographic and clinical data were presented as descriptive statistics (mean ± SD, median, IQR for continuous variables; counts and percentages for categorical variables).

We evaluated the association between HPV infection and selected behavioral and clinical variables (Table 3); however, no statistically significant associations were observed.

HPV vaccination showed no detectable effect on HPV positivity, likely due to the low number of vaccinated individuals in the cohort and short time since vaccination.

To improve reliability of analysis, the number of lifetime sexual partners was categorized into three groups. Results are shown in Table 3.

Chi-square test:

χ^2^ = 1.66;

*p* = 0.437.

No significant association was observed. Across the analyzed cohort of 400 women, HPV infections were detectable exclusively in cervical samples, with no HPV DNA identified in oropharyngeal swabs. The prevalence of HPV16, HPV18, and other high-risk genotypes in the cervix did not vary by age, and none of the assessed epidemiological or behavioral factors—including vaccination status, contraceptive method, or number of sexual partners—demonstrated a statistically significant association with HPV infection.

## 4. Discussion

The present study examined the prevalence and distribution of HPV16, HPV18, and other high-risk HPV genotypes in paired cervical and oropharyngeal samples from 400 sexually active women aged 20–40 years. The findings provide several important insights into the anatomical site specificity, epidemiology, and clinical relevance of HPV infections in young women from Central Europe.

The prevalence of high-risk HPV genotypes detected in cervical samples—9.5% for HPV16, 7.3% for HPV18, and 14.3% for other HR-HPV types—is consistent with previously reported prevalence rates in European populations of similar age. Several large-scale epidemiological studies, including those conducted in Eastern and Central Europe, have demonstrated comparable genotype distributions, with HPV16 as the most prevalent high-risk type and a substantial proportion of non-16/18 high-risk infections contributing to the overall burden of cervical HPV [5,12,16,25]. The relatively uniform distribution of HPV positivity across age groups in this cohort aligns with established epidemiological patterns showing that HPV acquisition peaks soon after sexual initiation and stabilizes during adulthood.

A key finding in this study is that none of the participants tested positive for HPV DNA in oropharyngeal swabs, despite the measurable prevalence of cervical infection. This observation reinforces the biological differences between the cervical transformation zone and the oropharyngeal mucosa. Several mechanistic studies have demonstrated that the oropharynx offers limited access to basal epithelial cells, supports lower viral persistence, and is characterized by slower epithelial turnover and a distinct immune environment [5,6,7]. As a result, high-risk HPV infections tend to be markedly less common in the oropharyngeal region, particularly among asymptomatic women. Our data fully support this paradigm and further suggest that the presence of cervical HPV infection does not correspond to detectable oral HPV involvement in young, immunocompetent women without risk factors.

We found no statistically significant associations between HPV infection and the evaluated clinical or behavioral variables, including age at sexual initiation, cytological abnormalities, contraceptive use, HPV vaccination status, and number of sexual partners. These findings may reflect the relatively homogeneous nature of the study population and the limited variability in these variables within this age group. Additionally, the absence of a detectable effect of vaccination may be related to limited vaccine uptake and short time since vaccination in this cohort—issues that have similarly affected real-world post-vaccination analyses in Europe [16]. The lack of association between number of sexual partners and HPV infection is also consistent with studies showing that sexual behavior interacts with numerous biological and immunological factors that modulate susceptibility to HPV, and simple exposure-based models may not capture individual variability.

Our findings corroborate previous studies demonstrating low prevalence of oropharyngeal HPV in women compared with men, and far lower than cervical HPV prevalence. Epidemiological data suggest that persistent oral HPV infection is uncommon in healthy, immunocompetent women and is often transient [4,5,6,7]. Studies focusing on asymptomatic adult women typically report oral HPV prevalence of 0.5–2%, consistent with the negative results observed in the present cohort. Furthermore, most reported oral infections in women occur in older age groups or populations with additional risk factors (smoking, alcohol, immunosuppression), none of which were assessed as major variables in this study.

This study reinforces the anatomical specificity of HPV infections and expands the availability of regional data from Central Europe—a region where the epidemiology of cervical HPV is known, but where matched cervical–oropharyngeal data remain scarce.

The complete absence of detectable oropharyngeal HPV infection in this cohort carries important clinical implications. These findings indicate that routine oropharyngeal HPV screening in asymptomatic young women is unlikely to provide meaningful information and is not supported by the observed data. Instead, HPV infections in this age group appear to remain localized to the cervical epithelium, emphasizing that cervical HPV testing should continue to serve as the primary preventive and diagnostic strategy. Genotype-specific monitoring, particularly for HPV16 and HPV18, remains relevant exclusively in cervical samples within this population. Consequently, these results reinforce the need to prioritize HPV-based cervical screening and vaccination programs, rather than extending surveillance to oral sites in low-risk women. From a public health perspective, the findings suggest that oropharyngeal sampling offers no additional value for screening programs targeting asymptomatic young women without risk factors for HPV-related oropharyngeal cancer.

This study has several important strengths, including its paired sampling design, which enables a direct and internally controlled comparison of cervical and oropharyngeal HPV prevalence within the same individuals. The large sample size (N = 400) increases the robustness and precision of prevalence estimates, while the clearly defined age range reflects a population of particularly high relevance for HPV acquisition, transmission dynamics, and post-vaccination epidemiology. The use of standardized, validated molecular detection methods ensures reliable identification of high-risk HPV genotypes. Moreover, the study provides valuable regional data, representing one of the few analyses from Central Europe to simultaneously evaluate HPV prevalence across two anatomical sites in healthy women.

## 5. Limitations

Several limitations should be acknowledged:

1. Cross-sectional design does not allow assessment of persistence vs. clearance of HPV infections.
2. Self-reported behavioral data (e.g., number of sexual partners) may be subject to reporting bias.
3. HPV vaccination coverage was low in the cohort, limiting analysis of vaccine impact.
4. Oropharyngeal sample collection methods vary across studies; although validated, swab-based methods may detect fewer low-level HPV infections compared with oral rinse protocols.
5. The study did not assess cofactors such as smoking, alcohol use, or immunosuppression, which could influence oral HPV susceptibility.

## 6. Future Directions

Future research should aim to build on these findings through longitudinal studies that assess HPV persistence and clearance over time, as cross-sectional analyses cannot distinguish between transient and sustained infections. Methodological refinement is also needed, particularly through multi-site sampling that combines swabs with oral rinse techniques to improve the sensitivity of oropharyngeal HPV detection. Future cohorts should include a larger proportion of vaccinated participants to enable meaningful stratification by vaccination status and evaluation of vaccine impact on genotype distribution. Additionally, incorporating lifestyle-related cofactors such as smoking and alcohol consumption would allow a more comprehensive assessment of risk determinants for both cervical and oropharyngeal HPV acquisition. Expanded genomic analyses—including viral load quantification, integration status, and host immune response markers—could further elucidate the biological underpinnings of infection dynamics. Finally, comparative studies involving male participants are essential to clarify sex-specific differences in oral HPV susceptibility and transmission patterns.

## 7. Conclusions

In conclusion, high-risk HPV infections in young women in this cohort were confined exclusively to the cervical epithelium, with no detectable oropharyngeal HPV DNA in any participant. The prevalence of HPV16, HPV18, and other high-risk genotypes was consistent with regional data, and none of the evaluated behavioral or clinical factors showed a significant association with infection. These findings underscore the importance of cervical HPV screening as the central preventive strategy and provide evidence that routine oropharyngeal HPV testing in asymptomatic young women is not clinically justified.

## Figures and Tables

**Table 1:** Selected demographic and clinical characteristics of the study population.

Variable	Age Category	N	%
age group	20–24	46	11.5
	25–29	146	36.5
	30–34	162	40.5
	35–40	46	11.5
place of residence	urban	295	73.8
	rural	105	26.2
education	higher education	203	50.7
education	secondary education	180	45.0
education	elementary education	17	4.2
marital/relationship status	in a stable partnership	161	40.2
	single	134	33.5
	married	105	26.2
smoking status	never smoker	226	56.5
	former smoker	93	23.2
	current smoker	81	20.2
parity	0	230	57.5
	≥1	170	42.5
number of HPV vaccine doses	unvaccinated	180	45.0
	3.0	118	29.5
	2.0	50	12.5
	1.0	29	7.2
	missing information	23	5.7
age (years)	Mean ± SD		29.6 ± 4.2
	Median (range)		30.0 (20.0–40.0)

**Table 2:** Prevalence of HPV16, HPV18, and HR-HPV in cervical swabs by age group.

Age Group Years	N	HPV16-Positive (%)	HPV18-Positive (%)	Positive for Other HRHPVs (%)	Oropharyngeal Swabs
20–24	46	8.7	6.5	13.0	negative
25–29	146	9.6	7.5	14.4	negative
30–34	162	9.9	7.4	14.8	negative
35–40	46	8.7	6.5	13.0	negative

**Table 3:** Association between selected behavioral and clinical variables and HPV infection status.

Variable	Category/Measure	HPV-Negative n (%)	HPV-Positive n (%)	Statistical Test	Statistic	*p*-Value
Age at sexual initiation	Continuous	—	—	r_pb	0.071	0.159
Cytology abnormalities	Yes/No	—	—	χ^2^	0.989	0.320
HPV vaccination	Yes/No	—	—	χ^2^	0.000	1.000
Hormonal contraception	Yes/No	—	—	χ^2^	0.993	0.609
Hormonal IUD	Yes/No	—	—	χ^2^	1.030	0.597
Number of sexual partners	0–1	73 (71.6%)	29 (28.4%)	χ^2^	—	—
	2–4	131 (70.4%)	55 (29.6%)	-	-	-
	≥5	72 (64.3%)	40 (35.7%)	-	-	-

Values are presented as absolute numbers (n) and percentages calculated within each category where applicable. For variables assessed using correlation-based analyses or where detailed categorical distributions were not the primary focus, n (%) values are not reported.

## Data Availability

The data supporting the findings of this study are available on request from the corresponding author.
